# Hybrid Work in German Public Administration: Social Resources, Occupational Health Literacy and Work Design Competencies in Association with Work Engagement

**DOI:** 10.3390/bs15081123

**Published:** 2025-08-19

**Authors:** Tanja Wirth, Elisabeth Rohwer, Leonie Jaß, Volker Harth, Stefanie Mache

**Affiliations:** Institute for Occupational and Maritime Medicine (ZfAM), University Medical Center Hamburg-Eppendorf (UKE), 20459 Hamburg, Germany; wirth.ext@uke.de (T.W.); elisabeth.rohwer@uni-bremen.de (E.R.); l.jass@uke.de (L.J.); harth@uke.de (V.H.)

**Keywords:** hybrid work, remote work, face-to-face contact, job demands and resources, work engagement, occupational health literacy, work design competencies

## Abstract

Background: Since the COVID-19 pandemic, hybrid work models are on the rise in public administration in Germany. Hybrid work poses new challenges for employees. Face-to-face contact with colleagues at the office may be limited, potentially affecting social relationships at work. This study aimed to examine job demands and resources pertaining to social relationships between employees in public administration with low and high face-to-face contact with colleagues. Furthermore, associations between social and personal resources with work engagement and the moderating role of face-to-face contact were explored. Methods: A cross-sectional online survey was carried out in a German public administration. Validated instruments were used to measure job demands and resources regarding social relationships, occupational health literacy, work design competencies, and work engagement. Differences between employees with low and high face-to-face contact with colleagues were examined using Pearson’s chi-square test and Welch’s *t*-test. Multiple linear regression was used to analyze associations between social and personal resources and work engagement. Simple moderation analyses were carried out to explore the role of face-to-face contact. Results: Overall, 127 employees in public administration completed the questionnaire. Employees with low face-to-face contact with colleagues at the office reported significantly higher fear of missing out at work and lower team cohesion and empowering leadership. Team cohesion as well as work design competencies and occupational health literacy (subscale willingness/responsibility) were significantly positively associated with work engagement, but no moderation effect of face-to-face contact could be observed. Conclusion: To reduce job demands regarding social relationships and strengthen social resources of hybrid workers, organizational measures could be taken to foster regular face-to-face contact with colleagues (e.g., overlapping attendance days). Additionally, training programs on work design competencies and occupational health literacy could positively impact employees’ work engagement.

## 1. Introduction

In the past, working in public administration in Germany was characterized by a high level of physical presence in the office (Siegel et al., 2020). Common reasons for this culture of presence were an inadequate technical infrastructure (Gelep et al., 2021; Neumann et al., 2020), the (hierarchical) leadership style (Neumann et al., 2020), and strict regulations for working from home, which was often limited to employees who cared for children or dependents or who had a long commute to the office (Gelep et al., 2021). This culture has changed rapidly since the COVID-19 pandemic. In a survey conducted among employees of four public administration organizations in Germany (N = 1189), 84% reported that, prior to the first COVID-19 lockdown in autumn 2020, they had worked exclusively on-site and had never engaged in remote work. This proportion decreased to 27% during the lockdown (Siegel et al., 2020). In the winter of 2021/2022, 63% of employees surveyed from municipal administrations in North Rhine-Westphalia, Germany (*N* = 838), reported that they have the possibility to work remotely, and 35% even worked more than half of their working hours from home (Niehaves et al., 2022). Thus, hybrid working models are on the rise in public administration. Hybrid work represents a mixture of on-site and remote work and is, therefore, flexible in terms of the place of work (Grzegorczyk et al., 2021; Quatram & van Kempen, 2021). However, this flexibility poses new challenges for organizations, such as finding agreements on the ways of working, coordinating hybrid work, and aligning office design and meeting culture with hybrid formats. Employees can also face new job demands and resources. For example, they need to find the right balance between working from home and the office, or they can face feelings of exclusion when they are not in the office, as well as invisible groupings between office and remote workers that can negatively impact social relationships at work (Babapour Chafi et al., 2022). To the best of our knowledge, little is currently known about specific job demands and resources in the context of the new work situation in public administration in Germany. Therefore, the present study aimed to examine the experiences of employees in public administration with hybrid work, as well as job demands and resources pertaining to social relationships, according to their amount of face-to-face contact with colleagues. Furthermore, associations between social and personal resources with work engagement and the moderating role of face-to-face contact were explored.

## 2. Theoretical Background and Hypotheses

### 2.1. Job Demands–Resources Model

The Job Demands–Resources (JD-R) model of Demerouti et al. (2001) served as the theoretical model for this study. The JD-R model differentiates between job demands and job resources. Job demands represent physical, psychological, social, or organizational aspects of the job that require sustained effort, such as an unfavorable work environment, and are associated with job strain (health impairment process). Job resources represent functional physical, psychological, social, or organizational aspects of the job, such as autonomy or feedback. According to the JD-R model, job resources can stimulate personal development and lead to high work engagement (motivational process) (Bakker & Demerouti, 2007; Demerouti et al., 2019). The JD-R model was later expanded by personal resources (Xanthopoulou et al., 2007), which can be defined as “aspects of the self that are generally linked to resiliency” (Hobfoll et al., 2003, p. 632). They contribute to explaining motivational outcomes and, therefore, play a similar role as job resources (Demerouti et al., 2019). This study focused on job demands and resources regarding social relationships, personal resources, and especially the motivational process of the JD-R model.

In the specific context of public administration, characterized by hierarchical structures, formalized communication channels, and institutional constraints, the JD-R model offers a robust framework for examining how these unique organizational features shape the interplay between demands, resources, and employee engagement. The evolving nature of work towards hybrid arrangements introduces novel challenges and opportunities for both job and personal resources, making this theoretical lens particularly relevant.

### 2.2. Job Demands and Resources Regarding Social Relationships in the Context of Remote and Hybrid Work

Although literature shows a greater tendency towards a beneficial impact of hybrid and remote work on the well-being of employees, some of the main challenges of these forms of work concern social relationships at work, such as feelings of social isolation (Charalampous et al., 2019). Studies among employees of public organizations and services described feelings of loneliness, isolation, and disconnection from colleagues with respect to remote work during the COVID-19 pandemic (Babapour Chafi et al., 2022; Seinsche et al., 2023b). These challenges may be amplified in public sector contexts due to organizational characteristics such as rigid hierarchies and formal communication practices (Rainey, 2009), which can limit spontaneous interpersonal interactions compared to private sector settings (Belzunegui-Eraso & Erro-Garcés, 2020; B. Wang et al., 2021). Thus, hybrid work in public administration entails unique social demands that warrant targeted investigation.

In addition, concerns related to limited knowledge exchange were expressed (Babapour Chafi et al., 2022). On the one hand, German public service employees stated that there was more social exchange and interaction, e.g., in the form of small talk, unplanned communication, and passing on information, when working in the office (Seinsche et al., 2023a, 2023b). On the other hand, employees from geographically distributed teams saw some benefits in remote work in that they felt better cohesion because of the possibilities to meet online and perceived easier collaboration. However, further challenges of remote work were described as receiving limited feedback and difficulties in overcoming the fear of missing out at work (Babapour Chafi et al., 2022). Establishing relationships, ensuring their quality, and building trust were also more difficult when working from home (Babapour Chafi et al., 2022; Seinsche et al., 2023b). Several studies support the findings on difficulties with relationships (Juchnowicz & Kinowska, 2021; Knardahl & Christensen, 2022; Wontorczyk & Rożnowski, 2022). Among remote, hybrid, and on-site workers in southern Poland, hybrid workers assessed their possibilities of promoting positive interpersonal relationships at work as significantly lower than on-site workers (Wontorczyk & Rożnowski, 2022). Juchnowicz and Kinowska (2021) found that remote work significantly limited the perception of supportive relationships with superiors and co-workers in the workplace. This was found for participants who worked exclusively remotely and for hybrid workers, who worked remotely 1–2 days a week. However, no significant effects were observed for those working remotely less than once a week or 3–4 days a week. In contrast, Knardahl and Christensen (2022) described a monotonic dose–response relationship between the hours worked at home and co-worker support. The perceived co-worker support decreased steadily as the number of hours worked at home increased.

With regard to the state of research, differences in job demands and resources regarding social relationships between different forms of work can be assumed. In the present study, the amount of face-to-face contact with colleagues in the office (low: once a week or less vs. high: several times a week) served as a proxy for the form of work (on-site, hybrid, remote). In this respect, the following two hypotheses were made.

**H1.**  *There is a significant difference in the amount of face-to-face contact with colleagues at the office between employees working predominantly at the office, working hybrid, and working predominantly remotely.*

**H2.**  *There is a significant difference in the job demand, such as fear of missing out at work (H2a), and in the social resources, such as team cohesion (H2b) and social support (H2c), between employees with low and high face-to-face contact with their colleagues at the office.*

### 2.3. Associations Between Social Resources, Personal Resources, and Work Engagement, as Well as the Moderating Role of Face-to-Face Contact

Work engagement was described as a distinct concept and defined as “a positive, fulfilling, work-related state of mind that is characterized by vigor, dedication, and absorption” by Schaufeli and Bakker (2004, p. 4). Vigor refers to high levels of energy and mental resilience as well as the willingness to invest effort in one’s work. Dedication means being strongly involved in one’s work and deriving a sense of significance from it. Absorption describes a state of being immersed and happily engrossed in one’s work (Schaufeli & Bakker, 2004). The main predictors of work engagement are job and personal resources (van den Heuvel et al., 2010).

One job resource that depicts social relationships and collaboration at work is team cohesion. Team cohesion can be understood as “the solidarity or unity of a group” (Forsyth, 2014, p. 10). Team cohesion highly correlates with concepts of social support at work and a sense of community (Lieb et al., 2024) and was found to be positively related to job satisfaction (Bae et al., 2023; Penconek et al., 2021). To the best of our knowledge, studies on the relationship between team cohesion and work engagement are still pending.

Hybrid work requires new forms of leadership that build on trust in the workforce and empower employees with more personal responsibility (Quatram & van Kempen, 2021). Empowering leadership is a leadership form that is characterized by the support of subordinates’ autonomy (Amundsen & Martinsen, 2014). Studies found statistically significant positive relationships between empowering leadership and work engagement (Forster & Koob, 2023; Tuckey et al., 2012).

In public administration, where institutional trust and service quality are paramount, social resources such as team cohesion and empowering leadership are critical not only for employee motivation but also for maintaining organizational legitimacy (Brewer, 2003; Zubair et al., 2021).

Under hybrid work conditions, these social resources might be disrupted, heightening their significance.

No studies in the field of public administration were identified. Therefore, we examined the following hypothesis.

**H3.**  *The social resources, such as team cohesion (H3a) and empowering leadership (H3b), are significantly associated with work engagement among employees in public administration.*

Working remotely from home places additional demands on employees compared to on-site work, such as high levels of self-discipline, personal responsibility, and self-organization (Quatram & van Kempen, 2021). Skills that are required to cope with the demands of an independent work organization can be summarized as work design competencies (Dettmers & Clauß, 2018). They can be defined as “knowledge of how to design favorable working conditions that enable effective management of one’s own work tasks, while at the same time promoting motivation and reducing stress” (Dettmers & Clauß, 2018, p. 17). In former studies, work design competencies showed first significant positive relationships with work engagement (Dettmers & Clauß, 2018) and appeared to be relevant to work ability, which indicates employees’ current and future ability to work (Niebuhr et al., 2022).

Another individual competence of employees in the context of working life comprises occupational health literacy (OHL) (Ehmann et al., 2021). The concept of OHL includes the aspects of accessing, understanding, appraising, and applying health information with regard to safety and health at work (Ehmann et al., 2021; Jørgensen & Larsen, 2019; Rauscher & Myers, 2014). In a recent study, knowledge-/skill-based OHL was positively associated with work ability among participants in diverse small and medium-sized enterprises (Friedrich et al., 2024). However, to the best of our knowledge, research on OHL is still sparse. Individual competencies to manage one’s own health could be important personal resources, especially in new hybrid work contexts where employees are left to their own devices to a certain extent (Ehmann et al., 2021). Therefore, the following hypothesis was examined in this study.

**H4.**  *The personal resources, such as work design competencies (H4a) and OHL (H4b), are significantly associated with work engagement among employees in public administration.*

Previous research has shown that predictors of work engagement and well-being differ depending on work arrangement (remote, hybrid, or on-site) (Grobelny, 2023; Wontorczyk & Rożnowski, 2022). For instance, positive interpersonal relations predicted engagement more strongly among remote workers, while supportive leadership showed stronger associations for on-site employees (Wontorczyk & Rożnowski, 2022). Grobelny (2023) highlighted that the relationship between job resources and well-being varies across different team settings and suggested extending the Job Demands–Resources (JD-R) model by including team type as a contextual factor.

From a theoretical perspective, face-to-face interaction enhances social presence, mutual trust, and spontaneous informal exchange, which are crucial for the effective functioning of social resources like team cohesion and leadership [e.g., Social Presence Theory, Media Richness Theory]. In physical work environments, social cues such as body language, tone of voice, and informal encounters are more salient, enabling stronger interpersonal bonds. Thus, face-to-face contact can be expected to amplify the effects of social resources on employee engagement.

Given the formalized and hierarchical structure of public sector organizations (Rainey, 2009), face-to-face interaction may play a particularly important role in maintaining social resources such as team cohesion and leadership support. As a result, the positive effects of these resources on employee engagement may be more pronounced in the public sector than in less formal organizational contexts.

Based on these theoretical and empirical considerations, we formulated the following hypothesis:

**H5.**  *Face-to-face contact with colleagues significantly moderates the relationship between the social resources, such as team cohesion (H5a) and empowering leadership (H5b) and work engagement.*

In contrast to social resources, personal resources such as work design competencies are internal, self-regulatory capacities that enable individuals to actively shape their work environment and maintain motivation and engagement, regardless of external conditions. According to the JD-R model, personal resources function as relatively stable individual characteristics that are not bound to situational factors like physical proximity to colleagues.

Moreover, personal resources rely less on interpersonal interaction and more on individual agency, problem-solving, and proactive behavior. These qualities can be deployed both in remote and in on-site work contexts. Thus, the positive relationship between personal resources and work engagement is expected to remain stable, independent of face-to-face work conditions.

Consequently, the relationship between personal resources and engagement is expected to be stable and not moderated by face-to-face contact.

**Figure 1:**
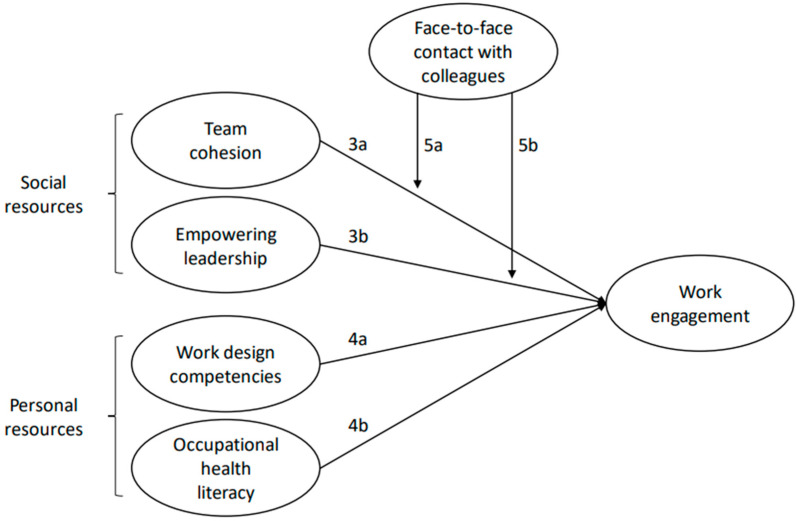
Conceptual Model of Hypotheses H3–H5: Associations Between Social and Personal Resources, Work Engagement, and the Moderating Role of Face-to-Face Contact.

Figure 1 visually summarizes the hypotheses H3-H5 on the associations between social and personal resources and work engagement and the moderation effects of face-to-face contact.

## 3. Materials and Methods

### 3.1. Study Design and Participants

A cross-sectional online survey was carried out in the public administration of a large city in Germany. Hybrid work employees and managers with at least one year of professional experience and a weekly working time of at least 19 h/week were eligible to participate in the study. Data collection took place between the end of August and the end of September 2023. The study was introduced to department managers and the staff council of the public administration in virtual meetings. The study aims, the content of the questionnaire, the target group, and the organizational procedure were explained, and open questions were clarified. Information material on the study and the link to the questionnaire were also made available. Department managers then forwarded the information and the link to their employees via email and invited them to participate in the study. Participation in the study was voluntary. Considering a multiple linear regression model with five predictors that was planned for the main analysis, a determination coefficient of *f*^2^ = 0.15 (medium effect), a statistical power of 0.90, and a significance level of α = 0.05, a total sample size of *n* = 116 was required (calculated with G*Power version 3.1.9.6).

### 3.2. Measures

#### 3.2.1. Sociodemographic Information and Form of Work

Sociodemographic information of study participants included age, gender, highest educational qualification, years of work experience, working time (hours/week), and leadership position. Further questions covered participants’ forms of work. One question asked about their experience with remote work, defined as working from home, while traveling, in cafés, or co-working spaces. Another question asked where participants usually work (“always at the office”, predominantly at the office”, “half of the time at the office, the other half of the time elsewhere”, “predominantly remote”, or “always remote”). In this context, it was also asked how often they meet their closest colleagues on-site at the company office. Possible answers were “never”, “about once a quarter”, “about once a month”, “about once a week”, “several times a week”, and “every day”. For analysis purposes, this face-to-face contact with colleagues was dichotomized into low (once a week or less) and high contact (at least several times a week). Finally, participants were asked which specific tools (“telephony”, “video conference tools”, “chats”, “emails or fax”, “letters”, or “others”) they used for their hybrid work with colleagues.

#### 3.2.2. Job Demands Regarding Social Relationships

Fear of missing out at work was assessed using the scale of Budnick et al. (2020). It describes the fear of missing out on opportunities, such as acquiring valuable information and building professional relationships, when one is absent or disconnected from work. The scale comprises ten items (e.g., “I worry that I might miss important work-related updates.”) that are measured on a 5-point Likert scale ranging from 1 = strongly disagree to 5 = strongly agree. The overall scale has shown high reliability (Cronbach’s alpha = 0.95) (Budnick et al., 2020).

#### 3.2.3. Job Resources Regarding Social Relationships

Team cohesion was measured using the subscale cohesion of the Questionnaire on Teamwork, developed by Kauffeld and Frieling (2001). The subscale reflects the cohesion of the group, the trusting and open interaction with each other, the social support, and the sense of unity in the team. It comprises eight items that consist of a pair of opposites, with a positive (e.g., “We talk openly and freely with each other.”) and a negative statement (e.g., “We don’t talk openly and freely with each other.”) on each side. There are six ratings to choose from between these two anchoring statements. Higher values indicate greater team cohesion. The scale has shown high reliability with Cronbach’s alpha of 0.87 to 0.90 in the validation study (Kauffeld & Frieling, 2001).

Social support was measured using the subscale social support from colleagues of the German version of the Copenhagen Psychosocial Questionnaire III (Lincke et al., 2021). The subscale assesses employees’ subjective impression of receiving support from colleagues if needed. It consists of two items measured on a 5-point Likert-scale ranging from 1 = always to 5 = never/almost never (e.g., “How often do you get help and support from your colleagues if needed?”). For the analysis, the five answer options were reversed and scored with 0, 25, 50, 75, and 100 points. Therefore, higher values indicate more social support. The two-item version of the subscale has shown high reliability (Cronbach’s alpha = 0.87) in a large international validation study (Burr et al., 2019).

Empowering leadership was assessed using the Empowering Leadership Scale (ELS) of Amundsen and Martinsen (2014), which consists of the two dimensions “autonomy support” and “development support”. Autonomy support (twelve items) includes empowering behaviors by the leader through delegation, coordination, and information sharing, encouraging initiative and goal orientation, efficacy support, and inspiring communication. An example is: “My leader conveys that I shall take responsibility”. Development support (six items) involves behavior on the part of the leader that promotes continuous learning and development of employees through their role modelling and guidance. An example item is: “My leader lets me see how he/she organizes his/her work”. Both dimensions are measured on a 7-point Likert-scale ranging from 1 = never to 7 = always. Validity and reliability of the ELS were confirmed in three studies (Amundsen & Martinsen, 2014).

#### 3.2.4. Personal Resources

To measure work design competencies, an 11-item scale developed by Dettmers and Clauß (2018) was used. It comprises the competencies of work planning, self-motivation, and stress avoidance (e.g., “When you think about your work, how well do you manage to structure your tasks by yourself?”). Items are assessed on a 7-point Likert scale ranging from 1 = very bad to 7 = very good. Validity and reliability (Cronbach’s alpha = 0.92) were proven for the overall scale (Dettmers & Clauß, 2018).

OHL was measured using the Occupational Health Literacy Scale (OHLS) (Friedrich et al., 2022; Friedrich et al., 2023). The OHLS comprises two subscales. The first assesses knowledge and skill-based processing of health information with eight items (e.g., “In your opinion, how easy or difficult is it to find easily comprehensible information regarding safety and health at the workplace?”). It uses a 4-point Likert scale ranging from 1 = very difficult to 4 = very easy. The second measures willingness and responsibility for occupational health using four items (e.g., “I find it very important to regularly inform myself about the rules of conduct regarding health and safety at my workplace.”). Response options range from 1 = strongly disagree to 4 = strongly agree. Both scales were validated and showed good reliability (Cronbach’s alpha = 0.88 and 0.74). Mean scores of the scales were calculated when at least 75% (6/8 and 3/4) of items were answered by participants. All scores were transformed according to the formula published by Friedrich et al. (2023) and interpreted as inadequate (0–25), problematic (>25–33), sufficient (>33–42), and excellent OHL (>42–50).

#### 3.2.5. Outcome Variable

Work engagement was assessed using the German short version of the Utrecht Work Engagement Scale (UWES-9) (Schaufeli & Bakker, 2004). It measures vigor, dedication, and absorption with nine items on a 7-point Likert scale ranging from 0 = never to 6 = always (e.g., “At my work, I feel bursting with energy.”). The UWES-9 was validated in ten countries, including Germany, and Cronbach’s alpha varied between 0.85 and 0.92 across the countries (Schaufeli et al., 2006).

### 3.3. Statistical Analyses

All participants who did not meet the inclusion criteria and who terminated the questionnaire early (before the actual scales) were removed from the dataset. The dataset was further checked for plausibility. The online questionnaire did not use forced answering. Therefore, missing values occurred among the individual variables, ranging from 0% to 15% (empowering leadership). For the description of the study sample, frequencies, means, and standard deviations (*SD*) were calculated. Internal consistencies of scales (Cronbach’s alpha) and correlations between variables were examined.

To analyze group differences between participants with low and high face-to-face contact with colleagues, Pearson’s chi-square test was carried out for categorical variables (H1). No expected cell frequencies were below 5. Cramér’s V served as a measure for the effect size and was interpreted as V = 0.1 (small), V = 0.3 (medium), and V = 0.5 (large) (Cohen, 1988, pp. 224–225). For group comparisons of metric variables (H2), it was examined whether a MANOVA could be used. However, prerequisites for normal distribution, linearity, homogeneity of variance, and homogeneity of covariance matrices were not met. Therefore, Welch’s *t*-test for independent samples with Holm–Bonferroni corrected alpha levels for multiple comparisons was applied, which is robust against unequal variances (Delacre et al., 2017; Rasch et al., 2011). Additionally, the group sizes were sufficiently large (*n* > 30 participants) so that the *t*-test could also be considered to be robust to the normal distribution (Bortz & Schuster, 2010; Herzog et al., 2019). Missing values were deleted pairwise. Cohen’s *d* was calculated and interpreted as *d* = 0.2 (small), *d* = 0.5 (medium), and *d* = 0.8 (large) (Cohen, 1988, pp. 25–26).

Associations between resources and work engagement (H3 and H4) were examined by hierarchical multiple linear regression. Prerequisites for the regression model were tested and met. A complete case analysis was conducted. Model 1 included social resources as predictors. Model 2 additionally included personal resources. The variables gender, work experience, and leadership position were considered as control variables. There was no substantial change in the standardized beta coefficients of the predictors with the control variables. Therefore, a final model without the control variables was applied and reported as suggested by Becker et al. (2016). Robust confidence intervals (CIs) and standard errors (SEs) were calculated using bias-corrected and accelerated (BCa) bootstrapping based on 5000 bootstrap samples. All analyses were carried out using IBM SPSS Statistics (version 26).

Additionally, two simple moderation analyses were carried out to examine the moderating role of face-to-face contact on the relationships between team cohesion and work engagement and empowering leadership and work engagement (H5).

Face-to-face contact was included as a moderator variable in the analysis to examine its potential influence on the relationship between resources and work engagement. For the purpose of statistical analysis and theoretical clarity, this variable was dichotomized. Participants were categorized into two groups based on their responses: those who reported working predominantly or entirely on-site were assigned to the “high face-to-face contact” group, while those who reported working mainly or exclusively remotely were assigned to the “low face-to-face contact” group.

This decision was based on both theoretical considerations and empirical distribution. From a theoretical standpoint, the distinction between frequent and infrequent in-person interaction reflects meaningful differences in the experience of hybrid work environments, particularly with regard to the accessibility of social resources. Empirically, the distribution of responses suggested a natural divide that supported the binary classification.

While we acknowledge that dichotomization can reduce variance and statistical power and obscure more subtle effects, this approach was chosen to facilitate interpretability and align with prior research in the field. The implications of this decision are further addressed in the discussion section.

Predictors and the moderator were mean centered for the analyses, robust coefficients and standard errors were calculated by using 5000 bootstrap samples, and heteroscedasticity-consistent standard errors (HC3) were retrieved. Moderation analyses were undertaken with the PROCESS macro v4.2 for SPSS (Hayes, 2022).

A post hoc power analysis was conducted using G*Power to assess the achieved statistical power for the moderation analysis.

## 4. Results

### 4.1. Characteristics of Study Participants

Overall, 302 employees in public administration were invited to participate in the online survey. Of them, 127 completed the questionnaire (response rate: 42.1%). The majority of participants were female (53%), held a master’s degree/diploma (47%), and worked 35 h per week or more (80%). About 20% had a leadership position. Table 1 displays the sociodemographic information of the study participants.

### 4.2. Study Participants’ Forms of Work

Most of the study participants worked either hybrid (40.2%) or predominantly from home (37.8%). Face-to-face contact with colleagues at the office at least several times a week was reported by 58.3%. At the time of the survey, the majority of participants indicated experiences with remote work of 3–5 years (40.2%). Nearly all participants used video conference tools, emails/fax, and telephony for hybrid work (all > 80%). All descriptive results on participants’ form of work and their experiences with remote and hybrid work are shown in Table 2.

Of the participants who worked predominantly at the office, 14.3% reported having low face-to-face contact with colleagues (once a week or less) and 85.7% reported having high contact (at least several times a week). Among those who worked hybrid, a comparatively larger proportion of 43.1% stated to have low contact, and 56.9% exhibited high contact. The majority of participants who worked predominantly remotely had low face-to-face contact with colleagues (56.3%), and 43.8% had high contact. The differences between the forms of work were statistically significant (*χ*^2^(2) = 12.88, *p* = 0.002). A medium effect was observed (Cramér’s V = 0.318). Therefore, hypothesis H1 was supported.

### 4.3. Descriptive Statistics of Study Variables

The internal consistencies of the main variables were good (Cronbach’s alpha > 0.8). Only the subscale willingness/responsibility of the OHLS showed a lower Cronbach’s alpha of 0.69, which was still at the threshold of an acceptable value (Kline, 2000) (Table 3).

The variable social support from colleagues showed a ceiling effect with a mean value of 86.12 (*SD* = 15.84) and a proportion of 45.7% of participants who reported the highest possible value of 100.

The majority of participants had an inadequate (35.4%) or problematic (29.9%) OHL when it came to knowledge and skill-based processing of health information. Only 4.7% reached excellent OHL in this respect. OHL with regard to willingness and responsibility for occupational health was inadequate among 37% of participants. Another 15.7% had problematic, 38.6% sufficient, and 8.7% excellent OHL on this subscale.

### 4.4. Associations Between Social and Personal Resources and Work Engagement

The social resources team cohesion (*r* = 0.367; *p* < 0.001) and empowering leadership (*r* = 0.323; *p* < 0.01) were both significantly and positively correlated with work engagement. The personal resources, such as work design competencies (*r* = 0.440; *p* < 0.001), OHL (knowledge-/skill-based) (*r* = 0.188; *p* < 0.05), and OHL (willingness/responsibility) (*r* = 0.266; *p* < 0.01), were also significantly positively correlated with work engagement (Table 4).

In order to control for potential confounding effects, the demographic variables of age, gender, and professional experience were included as control variables in the regression analyses. However, none of these variables showed a statistically significant association with the dependent variable of work engagement, nor did they substantially alter the effects of the main independent variables.

In the first regression model, team cohesion (*β* = 0.318; *p* = 0.001) and empowering leadership (*β* = 0.212; *p* = 0.028) were statistically significantly positively associated with work engagement. Both social resource variables together explained 17.5% of the variance in work engagement (*adj. R*^2^ = 0.175).

When personal resources were added to the regression model (model 2), the association between empowering leadership and work engagement was no longer statistically significant. Team cohesion (*β* = 0.247; *p* = 0.008) as well as the personal resources work design competencies (*β* = 0.345; *p* < 0.001) and OHL (willingness and responsibility) (*β* = 0.225; *p* = 0.011) were all statistically significantly positively associated with work engagement in the second model. Knowledge-/skill-based OHL showed no statistically significant association with work engagement. The variables of model 2 explained about 31.8% of the variance in work engagement (*adj. R*^2^ = 0.318) (Table 5). Thus, the hypotheses H3a and H4a were supported by the results, while hypothesis H3b was not. Hypothesis H4b was only partly supported for OHL (willingness/responsibility), but not for OHL (knowledge-/skill-based).

### 4.5. The Moderating Role of Face-to-Face Contact

A first moderation analysis was carried out on the moderating role of face-to-face contact on the relationship between team cohesion and work engagement (*F*(3, 114) = 10.34, *p* < 0.001, *n* = 118). Face-to-face contact did not statistically significantly moderate the relationship between team cohesion and work engagement (Δ*R*^2^ = 0.0093, *F*(1, 114) = 1.29, *p* = 0.259) (Table 6). Therefore, hypothesis H5a was rejected. The second moderation analysis examined the moderating role of face-to-face contact on the relationship between empowering leadership and work engagement (*F*(3, 101) = 13.02, *p* < 0.001, *n* = 105). There was no statistically significant moderation effect of face-to-face contact (Δ*R*^2^ = 0.0119, *F*(1, 101) = 1.59, *p* = 0.211) (Table 7). Thus, hypothesis H5b was also rejected.

The post hoc power analysis indicated that, with a sample size of *n* = 104, an alpha level of 0.05, and assuming a small-to-medium interaction effect (*f*^2^ = 0.06), the achieved power was approximately 0.68, which falls below the commonly recommended threshold of 0.80. This indicates an increased risk of a Type II error, i.e., failing to detect an existing interaction effect due to insufficient statistical power.

## 5. Discussion

This study is currently one of the few studies that examined working conditions among public administration employees in Germany and included the impact of new forms of work, such as hybrid work. Employees with low face-to-face contact with colleagues reported higher fear of missing out at work and lower team cohesion and empowering leadership. Additionally, team cohesion, work design competencies, and OHL with regard to willingness and responsibility were significantly associated with work engagement, but no moderation effect of face-to-face contact could be observed.

The results of this study underline the change of work forms in public administration towards more hybrid and remote work. About 80% of participants reported working either hybrid or predominantly remotely. This is in line with previous studies, which showed that a large proportion of employees in public administrations received more opportunities to work remotely during the COVID-19 pandemic (Niehaves et al., 2022; Siegel et al., 2020). However, our results also indicate that hybrid and remote work went along with lower face-to-face contact with colleagues at the office compared to on-site work. This is comparable to qualitative studies in which participants expressed feelings of disconnection from colleagues, less social interaction, and difficulties in establishing relationships (Babapour Chafi et al., 2022; Seinsche et al., 2023b). Our results further show that employees with low face-to-face contact with colleagues reported higher job demands and lower job resources with regard to social relationships at work. This concerned the fear of missing out at work, which was also described as challenging with respect to remote work in qualitative focus groups and interviews (Babapour Chafi et al., 2022). However, we are not aware of any quantitative study that has investigated this relationship to date and could confirm our findings. The perception of lower social resources among employees with low face-to-face contact was similarly observed among employees with increasing hours of work at home (Entgelmeier et al., 2023; Knardahl & Christensen, 2022). Assuming that hybrid and remote work correspond to less face-to-face contact, further studies confirmed fewer supportive relationships at the workplace in these forms of work (Juchnowicz & Kinowska, 2021; Wontorczyk & Rożnowski, 2022). However, a consistent dose–response relationship according to the time worked at home was not always apparent (Juchnowicz & Kinowska, 2021). This study cannot verify such a dose–response either due to the dichotomous categorization of face-to-face contact.

In accordance with the JD-R model, social and personal resources were positively associated with work engagement and contributed to an explained variance of medium height in the second regression model. Statistically significant results were observed for team cohesion, work design competencies, and OHL (willingness/responsibility). The association of team cohesion with work engagement is in line with former studies that found positive relationships between this social resource and work-related outcomes (Bae et al., 2023; Penconek et al., 2021). However, our results cannot confirm the positive association between the social resource empowering leadership and work engagement described in the literature (Forster & Koob, 2023; Tuckey et al., 2012). One reason could be that the sample may have been too small to detect an effect. In general, empowering leadership was perceived as lower by the participants in this study compared to the results of previous studies (Amundsen & Martinsen, 2015; Forster & Koob, 2023; Tuckey et al., 2012). This could be due to the typical hierarchical leadership structures in public administration (Niehaves et al., 2022). Because of hierarchical structures, responsibility is distributed vertically and employees have less autonomy and empowerment (Dreas & Klenk, 2021). With respect to personal resources, our results build on and confirm positive correlations between work design competencies and work engagement found in the validation study of the scale by Dettmers and Clauß (2018). Particularly, given increasing personal responsibility in the context of hybrid and remote work, expanding these competencies could, therefore, be beneficial. However, the causal relationship still needs to be verified in longitudinal studies. Regarding OHL, former studies often examined individual risk factors of OHL (Bassem et al., 2023; Torun, 2023; J. Wang et al., 2023). Studies focusing on the relationship between OHL and health- or work-related outcomes are rare (Friedrich et al., 2024). This study expands this knowledge by considering the relevance of OHL in the context of work engagement. While the subscale willingness/responsibility was positively associated with work engagement, no significant association was found for the subscale knowledge-/skill-based OHL. This could be explained by the different content of the scales. The willingness and responsibility to apply health-related information includes one’s own initiative to shape working conditions (Friedrich et al., 2023). It appears reasonable to assume that people who show this initiative also have a higher level of engagement with their work in general.

No moderation effect of face-to-face contact on the relationship between job resources and work engagement was found in this study. This contradicts previous studies that described different relationships of job resources with work engagement and workplace well-being among employees and teams working on-site, hybrid, or remotely (Grobelny, 2023; Wontorczyk & Rożnowski, 2022). One possible reason could be the use of face-to-face contact as a proxy for the different forms of work. To clarify the role of work forms of teams in the JD-R model (Grobelny, 2023), further investigations should follow.

### 5.1. Strengths and Limitations

A key strength of this study lies in its focus on a highly relevant and under-researched topic: the role of social and personal resources in shaping work engagement in the context of hybrid work within public administration. By applying rarely studied constructs such as occupational health literacy (OHL, (Ehmann et al., 2021)) using validated instruments, the study contributes meaningfully to the extension of the JD-R model into the public sector and hybrid work environments. Additionally, the online survey achieved a relatively high response rate of 42.1%, which exceeds typical response rates in comparable online employee surveys, particularly given the general decline in participation in questionnaire-based research (Köstner, 2022; Maurer & Jandura, 2009).

The study also benefits from the use of standardized procedures and psychometrically sound instruments. However, several limitations should be considered. First, a substantial amount of missing data (n = 23; 18.1%) in the regression model, resulting from the voluntary nature of item completion, meant that the required sample size determined by the a priori power analysis (n = 116) was not reached. Furthermore, the post hoc power analysis revealed that the final sample size of *n* = 104 did not reach the desired power level for detecting small-to-medium interaction effects. This underpowering may have contributed to the non-significant result in the moderation analysis, and the absence of a moderating effect of face-to-face contact should, therefore, be interpreted with caution.

In addition, the decision to dichotomize the moderator variable (face-to-face contact) may have reduced statistical power and obscured nuanced variance in the data. While this was performed to increase interpretability and comparability with prior studies, it nonetheless represents a methodological trade-off that may have contributed to the null finding.

As with all cross-sectional designs, the study does not permit conclusions about the directionality or causality of observed associations. Reverse causality and unmeasured confounders cannot be ruled out, limiting the explanatory power of the findings.

Moreover, the study’s generalizability is constrained by its sample, which was drawn from a single public administrative organization in one German city. Cultural and structural differences across organizations and regions may limit the transferability of the results to other public sector contexts. We explicitly acknowledge this and suggest that future research should replicate the study in broader and more diverse samples across different administrative settings and geographical locations.

Finally, a ceiling effect was observed for the variable social support, which may have attenuated the strength of observed group differences. Despite this, a statistically significant difference with a small-to-medium effect size was found, although it may underestimate the true effect.

Future studies employing longitudinal or experimental designs and larger, more diverse samples are needed to further validate and extend the findings presented here.

### 5.2. Implications for Future Research

The results of this study, especially regarding the associations between social and personal resources and work engagement as well as the role of face-to-face contact, should be verified with a larger study sample and in longitudinal studies. We used a cross-sectional approach within a not easily accessible and rarely studied group of employees in public administration that is characterized by a strong culture of physical presence in the office in the past. This study could now be extended to cover further public administrative organizations from other parts of Germany or to involve other sectors. In doing so, additional job demands and resources concerning the work organization, job content, or work environment in the context of hybrid work could be included that were not part of this study. Furthermore, it could be worthwhile to examine a possible dose–response relationship between the amount of face-to-face contact with colleagues and the job demands and resources pertaining to social relationships more closely. Because of the restricted sample size in this study, only dichotomous groups could be analyzed. However, a greater differentiation of face-to-face contact could allow for more precise statements about when these are associated with higher job demands and lower job resources.

### 5.3. Implications for Practice

This study showed that hybrid and remote work are related to lower face-to-face contact with colleagues at the office. In turn, employees with lower contact perceived higher job demands pertaining to social relationships and lower social resources. However, hybrid and remote forms of work also have a variety of advantages for employees, such as work flexibility and autonomy (Sampat et al., 2022), control over the work situation (Wontorczyk & Rożnowski, 2022), better work-–balance (Petscavage-Thomas et al., 2022; Sampat et al., 2022) and higher perceived productivity (Beno, 2021; Petscavage-Thomas et al., 2022), which were not part of this study. Therefore, organizations should not dispense with the option of these new forms of work. Nevertheless, the option to fully or partly work from home should be voluntary for employees (Sampat et al., 2022), and several measures could be taken to enhance social relationships at work. Concerning the results on employees’ fear of missing out at work, it is important that human resource professionals show an interest in the working conditions and career opportunities of hybrid workers, so that they do not feel left alone by their employers (Grzegorczyk et al., 2021). This also includes acknowledging that they have specific needs in terms of autonomy, (self-)management, and social relationships. Additionally, in hybrid team meetings with on-site and remote participants, special attention should be paid to involving remote participants in the discussions and decision-making processes (Grzegorczyk et al., 2021). In the context of team cohesion and social support, hybrid work arrangements with agreements between employers and employees on the flexibility of workspaces and working time (e.g., amount of time spent working remotely or at the office), as well as on fixed office days, could be useful (Grzegorczyk et al., 2021). In doing so, the participation of employees is very important with regard to their preferences for work days at the office (Sampat et al., 2022). Good organization and overlaps of the attendance days could considerably increase face-to-face contact among colleagues. Communication channels between hybrid team members, such as online consultation hours or short daily digital alignments, could simulate spontaneous communication when some members are not in the office (Grzegorczyk et al., 2021). In addition, further measures of team building should be implemented (Sampat et al., 2022), as the results of this study show that good team cohesion could also contribute to higher work engagement among employees. Although we could not find a positive association between empowering leadership and work engagement, it is well described that hybrid work requires a shift in management from input and process control to monitoring of goals and performance outcomes (Grzegorczyk et al., 2021; Niehaves et al., 2022; Quatram & van Kempen, 2021). This includes a leadership style that builds on trust in the workforce and more personal responsibility of employees (Dreas & Klenk, 2021; Quatram & van Kempen, 2021). A study among public service employees showed that participants perceived trust as an essential component of distant leadership and that it can be advantageous if supervisors implement a work culture based on work results (Seinsche et al., 2023b). One measure could be to make managers more aware of this type of leadership and to provide them with further training. In general, preventive measures on the individual level should be subordinate to measures on the organizational level. However, with regard to personal resources, training programs targeted at improving the ability to network through digital technologies and self-management skills of employees could also be useful (Grzegorczyk et al., 2021). This is particularly the case, as this study shows that work design competencies and willingness and responsibility for occupational health were associated with higher work engagement.

## 6. Conclusions

Hybrid forms of work have become an integral part of today’s working world. Therefore, it is important to understand specific job demands and resources of employees with regard to these new forms of work. This study can contribute to current research and practice by examining hybrid work in public administration. It shows the relevance of face-to-face contact in hybrid work with regard to employees’ fear of missing out at work, team cohesion, and social support from colleagues. The results imply that organizational measures should be taken to foster regular face-to-face contact among hybrid workers in public administration, as this could, on the one hand, reduce their job demands regarding social relationships and, on the other hand, strengthen their social resources at work. In addition, personal responsibility and self-management skills are crucial aspects of remote and hybrid work and of increasing importance with regard to a possible further expansion of these working models. Employees should, therefore, be supported in their competencies and personal resources. The results of this study suggest that it could be useful to install training programs that strengthen work design competencies and OHL of employees, as both were positively associated with work engagement. Only if organizations, supervisors, and employees consider the new job demands and resources that hybrid work entails, can this work be designed to promote health.

## Figures and Tables

**Table 1:** Sociodemographic information of study participants.

Variable	*n* = 127
Gender (*n* (%))	
Female	67 (52.8)
Male	49 (38.6)
Not specified/missing	11 (8.7)
Age, years (mean (*SD*), range)	46.2 (10.8), 18–65
Age, years (*n* (%))	
18–34	17 (13.4)
35–49	58 (45.7)
50–65	49 (38.6)
Missing	3 (2.4)
Highest educational qualification (*n* (%))	
Intermediate school-leaving certificate	17 (13.5)
General or subject-related qualification for university entrance	10 (7.9)
Bachelor’s degree/intermediate diploma	24 (18.9)
Master’s degree/diploma	59 (46.5)
Doctoral degree (or higher)	16 (12.6)
Missing	1 (0.8)
Work experience, years (mean (*SD*), range)	19 (11.4), 1–45
Work experience, years (*n* (%))	
1–5	18 (14.2)
6–15	40 (31.5)
16–25	27 (21.3)
>25	42 (33.1)
Working time, hours/week (mean (*SD*), range)	37.1 (4.9), 19–45
Working time, hours/week (*n* (%))	
<35 h/week	26 (20.5)
≥35 h/week	101 (79.5)
Leadership position (*n* (%))	
No	102 (80.3)
Yes	25 (19.7)

**Table 2:** Experiences of participants with remote and hybrid work.

Variable	*n* = 127
Form of work (*n* (%))	
Always at the office	0 (0)
Predominantly at the office	28 (22.0)
Hybrid (half of the time at the office, half of the time elsewhere)	51 (40.2)
Predominantly remote	48 (37.8)
Always remote	0 (0)
Face-to-face contact with colleagues (*n* (%))	
Once a week or less	53 (41.7)
At least several times a week	74 (58.3)
Experience with remote work (*n* (%))	
None	1 (0.8)
Several months	4 (3.1)
1–2 years	34 (26.8)
3–5 years	51 (40.2)
>5 years	37 (29.1)
Tools (programs, media) used for hybrid work (*n* (%)) ^1^	
Video conference tools	118 (92.9)
Emails or fax	109 (85.8)
Telephony	103 (81.1)
Chats	73 (57.5)
Letters (internal or federal mail)	4 (3.1)
Others (messenger services, SMS, cloud storage)	3 (2.4)

^1^ Multiple answers possible.

**Table 3:** Descriptive statistics of main variables.

Variable	*n*	Scale	Range	Mean (*SD*)	α
Fear of missing out at work	122	1–5	1–4.30	1.82 (0.71)	0.93
Team cohesion	125	1–6	1.75–6	4.48 (0.98)	0.87
Social support	127	0–100	25–100	86.12 (15.84)	0.81
Empowering leadership	108	1–7	1.61–6.83	4.27 (1.38)	0.96
Work design competencies	124	1–7	3–7	5.16 (0.86)	0.90
OHL (knowledge-/skill-based)	124	0–50	8.33–50	28.73 (8.07)	0.85
OHL (willingness/responsibility)	127	0–50	8.33–50	30.02 (9.45)	0.69
Work engagement	120	0–6	1.11–6	3.37 (1.21)	0.94

OHL = Occupational health literacy; α = Cronbach’s alpha.

**Table 4:** Correlation matrix of the main study variables.

Variable	1	2	3	4	5	6
1. Team cohesion	-	107	122	123	125	118
2. Empowering leadership	0.316 **	-	108	108	108	105
3. Work design competencies	0.265 **	0.186	-	121	124	119
4. OHL (knowledge-/skill-based)	0.159	0.331 ***	0.286 **	-	124	119
5. OHL (willingness/responsibility)	0.004	0.110	0.149	0.261 **	-	120
6. Work engagement	0.367 ***	0.323 **	0.440 ***	0.188 *	0.266 **	-

*n* is shown above the diagonal; Spearman’s correlation coefficients are shown below the diagonal: * *p* < 0.05 ** *p* < 0.01 *** *p* < 0.001 (2-tailed); OHL = Occupational health literacy.

**Table 5:** Regression analysis with work engagement as the dependent variable.

Variable	B (95%-CI) ^1^	*SE* ^1^	*β*	*p*
**Model 1**				
*Constant*	0.836 (−0.063 to 1.799)	0.481		0.082
Team cohesion	0.389 (0.156 to 0.616)	0.117	0.318	0.001
Empowering leadership	0.191 (0.024 to 0.350)	0.086	0.212	0.028
**Model 2**				
*Constant*	−1.840 (−3.120 to −0.511)	0.677		0.007
Team cohesion	0.302 (0.082 to 0.517)	0.112	0.247	0.008
Empowering leadership	0.136 (−0.027 to 0.298)	0.084	0.151	0.112
Work design competencies	0.508 (0.269 to 0.756)	0.123	0.345	<0.001
OHL (knowledge-/skill-based)	−0.009 (−0.046 to 0.03)	0.019	−0.055	0.648
OHL (willingness/responsibility)	0.030 (0.005 to 0.049)	0.011	0.225	0.011

*n* = 104; Model 1: *F*(2, 101) = 11.90, *p* < 0.001, *R*^2^ = 0.191, *adj. R*^2^ = 0.175; Model 2: *F*(5, 98) = 10.63, *p* < 0.001, *R*^2^ = 0.352, *adj. R*^2^ = 0.318; OHL = Occupational health literacy; ^1^ Confidence intervals and standard errors based on 5000 bootstrap samples (BCa).

**Table 6:** Moderation analysis: face-to-face contact as a moderator of the relationship between team cohesion and work engagement.

Variable	B (95%-CI)	SE	*p*
**Model 1: Main effects only**			
*Constant*	3.338 (3.129 to 3.547)	0.106	<0.001
Team cohesion (centered)	0.317 (0.174 to 0.589)	0.105	0.0004
Face-to-face contact (centered)	0.635 (0.211 to 1.058)	0.214	0.0036
**Model 2: + Interaction term**			
Team cohesion (centered)	0.317 (0.174 to 0.589)	0.105	0.0004
Face-to-face contact (centered)	0.635 (0.211 to 1.058)	0.214	0.0036
**Team cohesion × Face-to-face**	0.242 (−0.181 to 0.664)	0.213	0.2591

*n* = 118; Model 1: *R*^2^ = 0.212; Model 2: Δ*R*^2^ = 0.009, *F*(1, 114) = 1.29, *p* = 0.259. Note: Confidence intervals and SEs based on HC3 robust standard errors. All predictors were mean centered before computing the interaction term.

**Table 7:** Moderation analysis: face-to-face contact as a moderator of the relationship between empowering leadership and work engagement.

Variable	B (95%-CI)	SE	*p*
**Model 1: Main effects only**			
*Constant*	3.376 (3.151 to 3.601)	0.114	<0.001
Empowering leadership (centered)	0.245 (0.086 to 0.404)	0.080	0.003
Face-to-face contact (centered)	0.910 (0.453 to 1.367)	0.230	<0.001
**Model 2: + Interaction term**			
Empowering leadership (centered)	0.245 (0.086 to 0.404)	0.080	0.003
Face-to-face contact (centered)	0.910 (0.453 to 1.367)	0.230	<0.001
**Empowering leadership × Face-to-face**	0.202 (−0.116 to 0.522)	0.161	0.211

*n* = 105; Model 1: *R*^2^ = 0.236; Model 2: Δ*R*^2^ = 0.012, *F*(1, 101) = 1.59, *p* = 0.211. Note: Confidence intervals and SEs based on HC3 robust standard errors. All predictors were mean centered before computing the interaction term.

## Data Availability

The datasets presented in this article are not readily available because of German data protection requirements. Requests to access the datasets should be directed to the corresponding author.
